# Rapid multiplex high resolution melting method to analyze inflammatory related SNPs in preterm birth

**DOI:** 10.1186/1756-0500-5-69

**Published:** 2012-01-26

**Authors:** Silvana Pereyra, Tatiana Velazquez, Bernardo Bertoni, Rossana Sapiro

**Affiliations:** 1 Departament of Genetics, School of Medicine, University of the Republic, Gral. Flores 2125, Montevideo, Uruguay; 2 Departament of Histology and Embryology, School of Medicine, University of the Republic, Gral. Flores 2125, Montevideo, Uruguay

## Abstract

**Background:**

Complex traits like cancer, diabetes, obesity or schizophrenia arise from an intricate interaction between genetic and environmental factors. Complex disorders often cluster in families without a clear-cut pattern of inheritance. Genomic wide association studies focus on the detection of tens or hundreds individual markers contributing to complex diseases. In order to test if a subset of single nucleotide polymorphisms (SNPs) from candidate genes are associated to a condition of interest in a particular individual or group of people, new techniques are needed. High-resolution melting (HRM) analysis is a new method in which polymerase chain reaction (PCR) and mutations scanning are carried out simultaneously in a closed tube, making the procedure fast, inexpensive and easy. Preterm birth (PTB) is considered a complex disease, where genetic and environmental factors interact to carry out the delivery of a newborn before 37 weeks of gestation. It is accepted that inflammation plays an important role in pregnancy and PTB.

**Methods:**

Here, we used real time-PCR followed by HRM analysis to simultaneously identify several gene variations involved in inflammatory pathways on preterm labor. SNPs from TLR4, IL6, IL1 beta and IL12RB genes were analyzed in a case-control study. The results were confirmed either by sequencing or by PCR followed by restriction fragment length polymorphism.

**Results:**

We were able to simultaneously recognize the variations of four genes with similar accuracy than other methods. In order to obtain non-overlapping melting temperatures, the key step in this strategy was primer design. Genotypic frequencies found for each SNP are in concordance with those previously described in similar populations. None of the studied SNPs were associated with PTB.

**Conclusions:**

Several gene variations related to the same inflammatory pathway were screened through a new flexible, fast and non expensive method with the purpose of analyzing their association to PTB. It can easily be used for simultaneously analyze any set of SNPs, either as the first choice for new association studies or as a complement to large-scale genotyping analysis. Given that inflammatory pathway is in the base of several diseases, it is potentially useful to analyze a broad range of disorders.

## Background

Complex traits like cancer, diabetes, obesity or schizophrenia arise from an intricate interaction of genetic and environmental factors. Genetic bases of complex disorders are usually difficult to determine due to the fact that environmental factors such as life style and their epigenetic consequences are molding the development of the disease. It is proposed that these factors mask the patterns of inheritance.

Preterm birth (PTB) is considered a complex disease, where genetics and environmental factors interact to lead to an undesirable effect which is the delivery of a newborn before 37 weeks of gestation [1,2]. PTB complicates many pregnancies and has for decades been strongly associated with increased risk of neonatal morbidity and mortality [3]. As a multifactorial trait, maternal stress, multiple pregnancies, exposure to toxics during pregnancy and genetic predisposition are well-known risk factors behind PTB [4]. It is also generally accepted that inflammation plays an important role in pregnancy and in PTB [5-7]. An altered inflammatory activity due to functionally relevant polymorphisms of the innate immune system may influence pathways leading to PTB and, therefore, impact on its frequency and/or severity.

Post genomic era and high-throughput techniques like genome-wide association studies (GWAS) yield information on millions of single nucleotide polymorphisms (SNPs) distributed across the human genome, to indentify candidate genes involved in complex diseases [8,9]. Later, fewer SNPs should be replicated in different populations to confirm the results, implying the need of a middle throughput platform to fulfill the study requirements [10]. In addition, clinical applications after a GWAS should comprise testing the candidate SNPs in a particular individual or a small group of people. Thus, there is a pressing need for multi-SNP analysis methods that can reveal system-level differences in candidate patients that are suspected of suffering a particular condition.

Several SNP genotyping techniques had been developed, such as PCR followed by restriction fragment length polymorphism (RFLP), direct sequencing, or PCR-based TaqMan chemistry. Some of these techniques are either expensive or permit only one SNP per assay, meaning there are laborious and time-consuming.

High-resolution melting (HRM) analysis is a new and rapid method for mutation detection, in which polymerase chain reaction (PCR) and mutations scanning are carried out simultaneously in a single procedure [11]. Genotyping by high-resolution amplicon melting is achieved with a post-PCR short melting step and uses a next generation saturating double-stranded DNA binding dye. The amplicon melting profile depends on its length, GC content, sequence characteristics and heterozygosity. This approach provides a simple, closed-tube detection that can easily detect heteroduplexes as well as homoduplexes based on their characteristic melting curve profiles [12].

In the present study, we used HRM analysis to simultaneously identify mutations in four genes involved on inflammatory pathways. Genotype data from preterm newborns were compared to data acquired from those born at term. In particular, quadruplex amplicon genotyping by HRM analysis was developed to rapidly identify the allele frequencies of four SNPs related to inflammatory response.

## Methods

### Study samples and DNA extraction

Subjects in this study were unrelated offspring of women receiving obstetrical care at the Pereira Rossell Hospital Center, Montevideo, Uruguay. Mothers with a history of drug abuse, chronic inflammatory diseases or twin pregnancy were excluded. The study protocol was approved by the School of Medicine's Ethics Committee of the University of the Republic, Uruguay. Mothers were inquired regarding sociodemographic characteristics, obstetric history, and data from the newborn through a specific designed questionnaire. Study population characteristics were previously detailed in Rey et al. [13]. Informed written consent was obtained from mothers prior to collection of biological material. Whole-blood samples from 56 subjects born at term and 53 born preterm were extracted and DNA isolated using DNeasy Blood and Tissue kit (Qiagen, Hilden, Germany). Each DNA sample was checked under an UV spectrophotometer (Biophotometa, Eppendorf, Hamburg, Germany).

### SNP selection, primer design and real time PCR conditions

In order to test the capability of HRM analysis to distinguish variable types of SNPs, four different candidate genes were chosen based of several criteria: (1) SNPs that are common in the study population (minor allele frequency around 5%-10%), bearing in mind that ethnic variation in allele frequencies does exist; (2) SNPs with potential functional consequences (functional SNPs) that are likely to alter the expression levels (regulatory SNPs) or protein folding structure (non-synonymous SNPs); (3) SNPs that capture the variation across a gene and can be used later to assign a haplotype [14] and (4) SNPs with a potential role in the inflammatory cascade as well as interaction to environmental factors. The selected SNPs were: rs4986790 (Toll like receptor 4 (TLR4) gene), rs16944 (interleukin 1β (IL-1β gene), rs1800795 (interleukin 6 (IL6) genes) and rs375947 (Interleukin 12 Receptor B (IL12RB) gene).

Several pairs of primer sets were designed for the aforementioned SNPs based in their flanking sequences using Primer3 tool (http://frodo.wi.mit.edu/primer3/). We created amplicons with non-overlapping melting temperatures and smaller than 250 pb, to have higher sensibility in the HRM analysis. Following Seipp et al. [15] recommendations some of the primers were designed with the inclusion of either GC- or AT-tails in order to modify the amplicon's melting temperature (Tm).

### Real time PCR and high-resolution-melting analysis

HRM analysis was performed on the Rotor-Gene 6000™ real-time instrument (Corbett Life Science, Sydney, Australia) with Eva Green, a saturating dye technology (Type-it HRM PCR Kit, Qiagen, Hilden, Germany).

All oligonucleotides were first tested in a single one-amplicon reaction and then quadruplex amplification was optimized. To set up the multiplex reaction, primer concentrations were determined empirically. First, each amplicon was amplified individually using the same PCR conditions to identify its Tm and efficiency in genotyping with HRM. Secondly, multiplex HRM analysis was performed in equimolar concentrations of the four pair of primers. At last, primer concentrations were adjusted according to the size of the peak obtained: primers yielding amplicons with lowest peaks were augmented and *vice-versa*. In this way, primer concentrations were adjusted to equalize signal from all amplicons.

Finally, PCRs were performed in 10 μl reaction volumes, containing 1X Qiagen HRM-typing buffer, 2.46 μM of each TLR4 gene primer, 0.46 μM of each IL6 gene primer, 0.26 μM of each IL1β gene primer, 0.9 μM of each IL12RB gene primer and 25 ng of template DNA. PCR reaction was carried out with an initial hold at 95°C for 5 min, followed by 35 cycles of 95°C for 15 s and 60°C for 1 min, and then HRM ramps were generated by acquiring fluorescence data at a temperature ramp from 72 to 88°C. Individuals with homozygous and heterozygous genotypes were included as controls in all experiments.

HRM curves were normalized and genotype was assigned according to HRM curve shape by the Rotor-Gene software and visual inspection. Melting curves observed were analyzed separately for each amplicon in the software to assign genotypes to samples.

### Restriction fragment length polymorphism (RFLP) and sequencing

To corroborate genotype assignment, we performed a blinded study based on a subset of samples genotyped with PCR-RFLP assays. rs4986790 was analyzed using mismatched primers as described by Lorenz et al. [16]. rs16944 was analyzed according to Di Giovine et al. [17]. rs1800795 was amplified using same specific newly designed primer set employed in the real time PCR with the following conditions: 94°C for 5 min, then 35 cycles of 94°C for 30 s, 62°C for 30 s, 72°C for 30 s, and a final extension at 72°C for 5 min. The products were digested with 5 U of Hin1II (Fermentas Inc.) in a final volume of 20 μl at 37°C for 2 h. For rs375947 a modified protocol of Lee et al. [18] was employed, consisting in a double digestion with 5 U of Hin1II at 37°C for 2 h followed by a digestion with 10 U of TaqI at 65°C for 12 h. In all cases fragments were analyzed by electrophoresis on a 1.5% high-resolution agarose gel and stained with ethidium bromide for visualization.

A random subset of PCR samples for the four genes was also sequenced using the BigDye Direct Sequencing kit (Applied Biosystem).

### Statistical analyses

All SNPs were tested for Hardy-Weinberg disequilibrium with an exact test. Case-control association tests were performed using *χ*^2 ^test and the significance of associations between genotypes and PTB were assessed using logistic regression. Statistical analyses were performed with PLINK v1.07 [19].

## Results

### HRM settings

**Figure 1:**
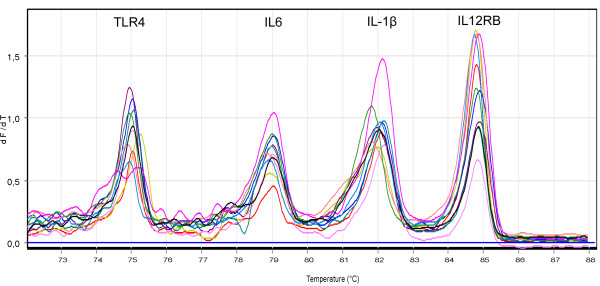
**Representative quadruplex melting curves obtained by HRM**. The melting peaks correspond to rs4986790 (TLR4), rs1800795 (IL6), rs16944 (IL-1β) and rs375947 (IL12RB) as temperature increases.

**Figure 2:**
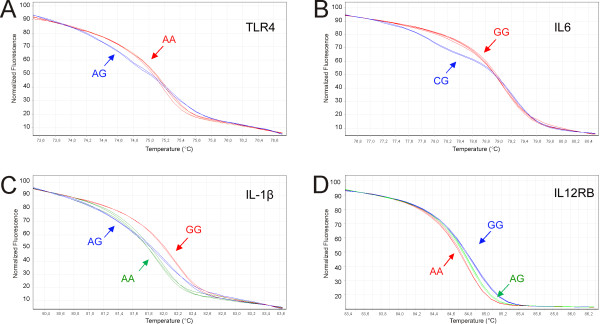
**Normalized HRM denaturation profile for all SNPs analyzed**. **A**) rs4986790 (TLR4). Red: Homozygote AA. Blue: Heterozygote AG. **B**) rs1800795 (IL6). Red: Homozygote GG. Blue: Heterozygote CG. **C**) rs16944 (IL-1β). Red: Homozygote GG. Blue: Heterozygote AG. Green: Homozygote AA. **D**) rs375947 (IL12RB). Red: Homozygote AA. Green: Heterozygote AG. Blue: Homozygote GG.

Designed conditions allowed obtaining a four-amplicon multiplex real-time PCR (Figure 1). The primers that best fitted conditions in order to obtain a four amplicon multiplex are indicated in Table 1. Temperature of melting (Tm) of amplicons is clearly separated from each other without overlap, spanning a 12°C temperature range. Amplicon with lowest Tm corresponds to rs4986790 (Tm = 75.5°C), in which we found the homozygous genotype AA and heterozygous AG (Figure 2A). Second amplicon is rs1800795 with Tm = 78°C and only homozygous GG and heterozygous CG genotypes were found (Figure 2B). For rs16944 (Tm = 83°C) (Figure 2C) and rs375947 (Tm = 85°C) (Figure 2D) all three genotypes where observed in each case.

**Table 1:** Primer sequences and amplicon information for the quadruplex HRM PCR assay

SNP reference number	Gene	Chromosome	Primer sequence (5'-> 3')		PCR product size (bp)	Primer concentration in multiplex PCR (μM)
rs4986790	TLR4	9	F	ATTTGACCATTGAAGAATTCCG	157	2.46
			R	TGTTGCCATCCGAAATTATAAG		
rs1800795	IL6	7	F	GCCTCAATGACGACCTAAGC	105	0.46
			R	GGGGCTGATTGGAAACCTTA		
rs16944	IL1β	2	F	CTTGGGTGCTGTTCTCTGCCTC	126	0.26
			R	CAACTCCGTCAGGAGCCTGAAC		
rs375947	IL12RB	19	F	CTGCCATTCAATGCAATACG	241	0.9
			R	CCCTGTAGGGTCAGGGGTAT		

### Genotype assignment

rs4986790, rs1800795, rs16944 and rs375947 genotypes were correctly assigned from HRM curves observed in the Rotor-Gene software, using sequenced samples included as control genotypes as reference for assignment in all runs. Only 2 samples out of 109 could not be confidently genotyped by multiplex HRM in the case of rs375947 (Additional file 1).

Allelic frequencies were calculated for each SNP (Table 2). Hardy-Weinberg (H-W) disequilibrium exact test indicates that SNPs rs16944, rs4986790, rs1800795 are in Hardy-Weinberg equilibrium (*p *> 0.05), whereas rs375947, located in IL12RB gene, is not. Particularly, if case and control groups are discerned, H-W disequilibrium test for rs375947 indicates control individuals are in H-W equilibrium, while case individuals are not (Table 2). No significant association was found between term and preterm newborns, for the analyzed SNPs (*p *> 0.05; Table 2).

**Table 2:** Allelic frequencies for each analyzed SNP in cases and controls.

SNP	Minor allele	MAF in cases	MAF in controls	P-values HWE	OR	P-values OR
rs4986790	G	0.0472	0.0179	1	2.81	0.2291
rs1800795	C	0.1698	0.1429	0.0691	1.29	0.5441
rs16944	A	0.3962	0.4018	0.4262	0.98	0.93
rs375947	G	0.3023	0.3571	0.0002	0.84	0.5232

Statistical significance for the Hardy-Weinberg test (p-value H-W equilibrium) and Odd Ratios (OR) based on a logistic regression. MAF: Minor Allele Frequency.

### Genotype verification

**Figure 3:**
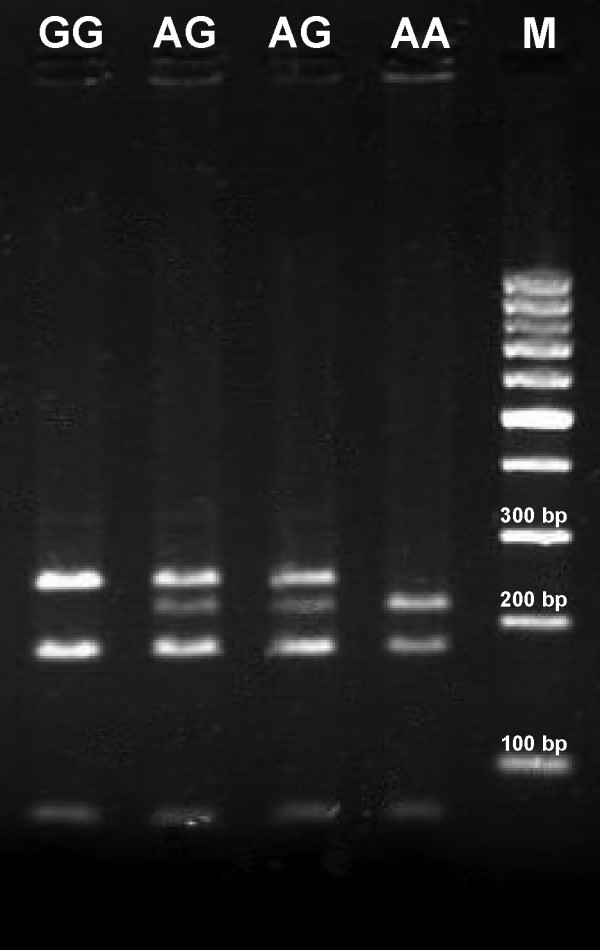
**Digested rs375947 (IL12RB) electrophoresis patterns for each genotype**. Genotypes are marked in each well. M: molecular weight marker.

The accuracy of the genotyping results was confirmed by RFLP and/or sequencing of the PCR product in a blinded study, for which random samples from our data study were used (Additional file 2). rs375947 (IL12RB) was assayed by means of a double digestion with restriction enzymes in a set of random individuals. The procedure was a modification of the strategy used by Lee et al. [18] and obtained a better discrimination of the genetic variations. The rs375947 amplicon is a 487 bp length fragment and was digested with Hin1II and TaqI. The TaqI restriction site is localized at 311 bp from the 5' end. TaqI restriction site is a non polymorphic restriction site that allows distinguishing the fragments generated by Hin1II. When the 487 bp fragment is digested by Hin1II, a non polymorphic restriction site is localized at 243 bp from the 5' end. A second Hin1II restriction site exists at 213 pb from the 5'end only at the SNP's mayor allele. After digestion with both enzymes, heterozygous SNPs yield a band pattern with 243, 216, 176, 68 and 27 bp fragments. Meanwhile, homozygotes are recognized because of the absence of the bands of 216 and 27 pb in the case of GG genotype (band pattern: 243 pb, 176 pb and 68 pb); or the absence of 243 bp in the case of AA (band pattern: 216 pb, 176 pb, 68 pb and 27 pb) (Figure 3).

### Conditions that affected the efficiency of the multiplex PCR

Design and concentration of primers proved to be the key step in this technique. Primers that generated amplicons between 50 to 250 bp and that were designed to avoid having more than one SNP per amplicon got the higher HRM efficiency. Efficiency is defined by the ability to discern and call different genotypes. Amplicons with no-overlapping Tm's are an absolute requirement to be able to distinguish between the variants in a multiplex HRM analysis. In order to modify an amplicon's Tm it is more effective to design primers with varying lengths than to design modified primers. The inclusion of GC- or AT-tails in the primers (as in Seipp et al. [15]) did not notoriously affect amplicon's Tm in this particular multiplex.

As expected, quality and concentration of the DNA sample influenced multiplex efficiency. Software genotype assignment tool reached nearly 100% of accuracy when DNA sample had an OD 260/280 ratio within the normal range (1.8-2.0) and a concentration of 50 ng/μL or higher. However, if some of these conditions were not met, the use of visual inspection of the curves allowed a correct assignment of the genotype in most cases.

Results can be viewed and genotype assigned as either a melting plot or a normalized difference plot as presented in the Rotor-Gene software, being the former one the most informative (Additional file 3).

## Discussion

In this study, we developed a quadruplex real-time HRM -PCR test which allows rapid and reliable SNP genotyping. Applying this technique, we were able to efficiently genotype variations in four inflammation-related genes at the same time in a case-control study population. The accuracy of the test was successfully verified through independent techniques such as PCR-RFLP and/or sequencing.

Multiplex HRM strategy has many important advantages. Unlike conventional genotyping methods, this assay does not require post PCR manipulations, saving time and reducing contamination risk. In addition, in quadruplex amplification such as the one designed here, assay costs are greatly reduced by analyzing four SNPs simultaneously. We have also showed that common oligonucleotide pairs can be used in this analysis, hence reducing optimization time. Thus, additional modifications to change amplicon's Tm, as proposed by Seipp et al. [15], are not needed. Primer design is a key step in this strategy. Recently, a multiple SNP HRM genotyping assay was presented, but the authors measured different SNPs in the same amplicon [20]. In our case, we specifically avoided more than one SNP in each amplicon. Potentially, two SNPs could give 9 different combined signals for the same amplicon, distorting the other SNP signals in a quadruplex study. There is a limited range to separate curves in HRM from different amplicons and four SNPs seems to be the present limit for this technology [21,22].

Rotor-Gene platform is commonly used to perform HRM analysis (e.g. [23,24]); however, no quadruplex amplification had been reported in this instrument. A quadruplex PCR was previously reported in the LightCycler-32 platform, but this could not be reproduced in other instruments [15]. Here, we have designed the first report of a multiplex HRM genotyping assay in a Rotor-Gene platform.

Genotyping was straightforward in all SNPs, with homozygous HRM profiles differing from heterozygous in curve shape, while the different homozygotes being easily distinguished because of their Tm shift.

Interestingly, in this study we were not able to find some of the variants already reported by other authors. Namely, we were not able to identify the minor homozygote genotype (CC) for rs1800795 (IL6) or the GG genotype for rs4986790 (TLR4) in our study population. It is reported that it may be difficult to distinguish common homozygotes from rare homozygotes in HRM for some SNPs [24], which could explain this situation. However, we independently assayed a subset of samples with PCR-RFLP and/or sequencing, finding most genotyping calls identical to those made based on HRM results, suggesting that those variants are more likely not present in the analyzed subjects. The absence of such genotypes could be attributed to the limited sample size in our analysis, but could also be explained by different allele frequencies present here in comparison to other populations. Previously, Rey et al. [13] screened over 400 patients from a Uruguayan sample genotyping SNP rs4986790 (TLR4) without finding the rare homozygote, indicating this genotype is present in low frequency in this population. On the other hand, HAPMAP data revealed that rs4986790 GG genotype is very rare in nearly all studied populations. Moreover, although the CC genotype for the rs1800795 (IL6) is present in 30% in the European population, it is absent in the Mexican population (Table 3). Uruguayan and Mexican populations have a similar genetic structure with contribution from Spaniards, Africans and Native Americans [25], which could be explaining the frequencies shown in Table 3. Taking all these data together, it is more likely that the absence of the rare homozygote in our study could be related to allele architecture in the population and not to HRM technical limitations.

**Table 3:** Genotype frequencies for each analyzed SNP in Europe (CEU), Japan (JPT), Yoruba (YRI), Mexican (MXC) populations and the Uruguayan sample *

Population	SNP	Gene	Genotype	Genotype	Genotype
	**rs4986790**	**TLR4**	**A|A**	**A|G**	**G|G**
CEU			0.933	0.067	
JPT			1		
YRI			0.933	0.067	
MXC			0.939	0.061	
This study			0.936	0.064	
	**rs1800795**	**IL6**	**C|C**	**C|G**	**G|G**
CEU			0.305	0.458	0.237
JPT					1
YRI					1
MXC				0.320	0.680
This study				0.312	0.688
	**rs16944**	**IL1β**	**A|A**	**A|G**	**G|G**
CEU			0.145	0.4	0.455
JPT			0.222	0.444	0.333
YRI			0.321	0.491	0.189
MXC			0.240	0.520	0.240
This study			0.138	0.523	0.339
	**rs375947**	**IL12RB**	**A|A**	**A|G**	**G|G**
CEU			0.383	0.483	0.133
JPT			0.364	0.5	0.136
YRI			0.567	0.4	0.033
MXC			0.720	0.280	
This study			0.541	0.259	0.200

* HapMap frequencies < 0.01 are not indicated

The developed assay focuses on variants related to inflammatory pathways, involved in host defense mechanisms, innate immunity activation and infection, as indicated in some of the web-based reference databases that link the genome to biologic systems (Kyoto Encyclopedia of Genes and Genomes, http://www.genome.jp/kegg/pathway.html; http://www.genome.jp/kegg-bin/show_pathway?map04620). For instance, the non-synonymous TLR4 SNP analyzed here (rs4986790) has been associated with differences in lipopolysaccharide responsiveness and predisposition to complex diseases like atherogenesis, respiratory dysfunction and preterm birth [26-29]. IL-6 -174 (rs1800795) G allele carriers produce higher levels of IL-6 than those with the CC genotype, and have a higher prevalence of systemic juvenile-onset chronic arthritis, lipid abnormalities and insulin resistance [30,31]. Also, analyzed SNP in the IL-1β gene promoter (rs16944) affects IL-1β expression and is associated with increased risk of cancer and preterm birth [32,33]. The protein encoded by IL12RB gene is a type I transmembrane protein that belongs to the hemopoietin receptor superfamily. The lack of expression of this gene was found to result in the immunodeficiency of patients with severe mycobacterial and *Salmonella *infections [34], and particularly, the rs375947 SNP is associated with atopic dermatitis [35]. Likewise, using other biological databases which identify SNPs associated to a particular condition, this multiplex HRM method could be potentially applied to analyze the risk to suffer the trait of interest of both patients and populations.

Although over the last years, high-resolution SNP genotyping arrays have greatly facilitated the identification of major genetic risk factors underlying complex diseases, such major genes seem to be rare. Instead, there is growing evidence that rare alleles and evolutionarily short-lived mutations play a major role in the etiology of complex disorders, which seem to be far more heterogeneous than previously assumed [36]. Dissection of complex disorders into many separate entities could greatly increase the chances for understanding the underlying pathogenetic mechanisms; for example, by defining novel candidate genes that are part of the same pathway [36]. Given that inflammation has been implicated in the mechanisms responsible for preterm and term parturition [5,6], screening of the chosen SNPs is a reasonable strategy to understand both mechanisms. Even more, inflammatory and immunological aspects are postulated to be on the base of several complex diseases others than PTB like atherogenesis, cancer, metabolic affections, endocrinophatys and even obesity [37-39]. Thus, as SNPs selected here are situated in genes related to an inflammatory pathway, we describe a potential tool to confident and rapidly screen gene variations that are common to a broad range of disorders.

Multiplex analysis by HRM could also be an accurate and reliable method for mutation detection in known genes after the identification of particular variations through the powerful existing strategies for elucidating genetic variations, such as array methods, whole-genome screening for copy number variation and high-throughput and low-cost sequencing.

## Conclusions

Several gene variations related to the same inflammatory pathway were screened with the purpose of analyzing their association to preterm birth. Multiplex HRM analysis has proved to be a flexible, fast and non expensive tool. It can easily be used for any set of SNPs, either as the first choice for new association studies or as a complement to large-scale genotyping analysis. It should be considered as a reliable genotyping technique for low or middle throughput projects. At last, the developed method can now be employed to assay genetic associations to preterm birth through a larger scale population study.

## List of Abbreviations

SNPs: single nucleotide polymorphisms; HRM: High-resolution melting; PCR: polymerase chain reaction; PTB: Preterm birth; GWAS: genome-wide association studies; RFLP: restriction fragment length polymorphism; TLR4: Toll like receptor 4; IL-1β: Interleukin 1β; IL6: Interleukin 6; IL12RB: Interleukin 12 Receptor B; H-W: Hardy-Weinberg; OR: Odd Ratios; MAF: Minor Allele Frequency; Tm: Temperature of melting; CEU: European population; JPT: Japanese population; YRI: Yoruba; MXC: Mexican population; ND: No data.

## Competing interests

The authors declare that they have no competing interests.

## Authors' contributions

SP and TV designed HRM conditions, primers, and carried on molecular genetics experiments. SP, TV, BB and RS were involved in data analysis. BB and RS designed the study, and participated in its design and coordination. SP and BB performed statistical analyses. SP, BB and RS wrote the manuscript. All authors contributed to and have approved the final manuscript.

## Supplementary Material

Additional file 1 **Genotype call by HRM for all newborns**. Multiplex HRM assigned data to all samples ND: no data. Click here for file

Additional file 2 **Subset of DNA samples genotyped by different techniques**. Genotype assignment for each analyzed SNP by Multiplex HRM, RFLP and sequencing. Click here for file

Additional file 3 **Figure S1**. A) Melting profile for rs16944 (IL1β). Red: Homozygote GG. Blue: Heterozygote AG. Green: Homozygote AA. B) Normalized difference plot for rs16944 (IL1β), genotype AG as a reference. Click here for file
