# Supplementary material for: Rapid multiplex high resolution melting method to analyze inflammatory related SNPs in preterm birth
Source: BMC Res Notes. 2012 Jan 26;5:69. doi: 10.1186/1756-0500-5-69 (PMC3298535; doi:10.1186/1756-0500-5-69)
Supplement: Additional file 1 — Genotype call by HRM for all newborns. Multiplex HRM assigned data to all samples ND: no data. [file 1756-0500-5-69-S1.PDF]

Additional file 1: genotype call by HRM for all newborns

Preterm group						Term group					
**Sample ID**	**Gestational Age (months)**	**Genotype rs4986790**	**Genotype rs1800795**	**Genotype rs16944**	**Genotype rs375947**	**Sample ID**	**Gestational Age (months)**	**Genotype rs4986790**	**Genotype rs1800795**	**Genotype rs16944**	**Genotype rs375947**
BP001	27	AA	GG	GG	AG	BC017	37	AA	GG	GG	AA
BP021	29	AA	GG	GG	AG	BC034	37	AA	GG	AG	AG
BP059	29	AA	CG	GG	AA	BC037	37	AG	GG	AA	AA
BP011	30	AA	CG	AG	GG	BC043	37	AA	GG	AG	AG
BP031	30	AA	CG	GG	AA	BC054	37	AA	CG	AG	AA
BP010	31	AA	GG	AG	AA	BC124	37	AA	GG	GG	AA
BC016	32	AA	CG	AG	GG	BC145	37	AA	CG	AG	AA
BP016	32	AA	GG	AG	GG	BC233	37	AA	GG	GG	AA
BP020	32	AA	GG	AG	AA	BC018	38	AA	GG	GG	AG
BP030	32	AA	GG	AG	AA	BC020	38	AG	GG	GG	GG
BP038	32	AA	GG	AG	AA	BC045	38	AA	GG	AG	GG
BC015	33	AA	CG	AA	GG	BC051	38	AA	GG	AG	AG
BP007	33	AG	GG	AG	AA	BC053	38	AA	GG	GG	GG
BP051	33	AA	CG	GG	AA	BC113	38	AA	GG	AG	GG
BP062	33	AA	CG	GG	AG	BP055	38	AA	CG	AA	AG
BP063	33	AA	GG	AA	GG	BP056	38	AA	GG	AG	AA
BC006	34	AA	GG	GG	AG	BC004	39	AA	GG	AG	AA
BC008	34	AA	GG	GG	AA	BC011	39	AA	GG	GG	AA
BC009	34	AA	GG	AA	AA	BC021	39	AA	GG	AG	GG
BC010	34	AA	GG	GG	AG	BC025	39	AA	GG	AG	AA
BP008	34	AA	GG	AG	AA	BC028	39	AA	GG	AG	AA
BP022	34	AA	CG	GG	AA	BC032	39	AA	GG	GG	AA
BP028	34	AA	GG	GG	AA	BC035	39	AA	CG	AA	AA
BP029	34	AA	GG	GG	AA	BC038	39	AA	GG	GG	AA
BP044	34	AA	CG	AA	AA	BC047	39	AA	GG	AG	AG
BP048	34	AA	GG	AA	AG	BC048	39	AA	GG	AA	AG
BP050	34	AA	GG	AG	AA	BC049	39	AA	CG	GG	AG
BP053	34	AA	GG	AA	AA	BC164	39	AA	CG	AG	AA
BP003	35	AA	GG	AG	AG	BC235	39	AA	CG	AG	GG
BP006	35	AA	GG	AG	AA	BC281	39	AA	CG	GG	AG
BP013	35	AA	GG	AG	AA	BP057	39	AA	GG	AG	AG
BP014	35	AG	CG	AG	AA	BC001	40	AA	GG	AG	AG

Preterm group (cont.)

Sample ID	Gestational Age (months)	Genotype rs4986790	Genotype rs1800795	Genotype rs16944	Genotype rs375947
BP023	35	AA	CG	AG	AA
BP036	35	AA	CG	AA	GG
BP040	35	AA	GG	AG	GG
BP041	35	AA	CG	AG	ND
BP043	35	AA	CG	AG	AG
BP047	35	AA	GG	GG	AA
BP060	35	AG	CG	AG	AA
BP061	35	AA	CG	GG	AG
BP082	35	AG	GG	GG	GG
BP009	36	AA	GG	AA	AA
BP017	36	AA	GG	AG	ND
BP024	36	AA	GG	GG	GG
BP025	36	AA	GG	AG	GG
BP026	36	AA	GG	GG	ND
BP034	36	AA	GG	GG	AA
BP039	36	AA	GG	AG	AA
BP042	36	AA	CG	AG	AG
BP045	36	AG	CG	GG	AA
BP046	36	AA	GG	AG	AA
BP049	36	AA	GG	AG	AA
BP054	36	AA	GG	AG	GG

Term group (cont.)

Sample ID	Gestational Age (months)	Genotype rs4986790	Genotype rs1800795	Genotype rs16944	Genotype rs375947
BC002	40	AA	GG	AG	AA
BC007	40	AA	GG	AA	AA
BC013	40	AA	GG	GG	GG
BC022	40	AA	CG	AG	GG
BC023	40	AA	CG	AG	AA
BC024	40	AA	GG	AG	AA
BC026	40	AA	CG	AG	AG
BC027	40	AA	GG	GG	AA
BC033	40	AA	CG	AG	AA
BC036	40	AA	GG	AG	AA
BC039	40	AA	GG	AG	AG
BC042	40	AA	GG	GG	AA
BC044	40	AA	GG	GG	GG
BC100	40	AA	GG	AG	AA
BC114	40	AA	GG	AG	AG
BC216	40	AA	GG	AG	AA
BC217	40	AA	CG	GG	AA
BP058	40	AA	GG	AA	AA
BC003	41	AA	GG	GG	AG
BC012	41	AA	CG	AG	AA
BC019	41	AA	CG	AG	AG
BC029	41	AA	CG	GG	AA
BC046	41	AA	GG	AG	AG
BC052	41	AA	GG	AA	AG
