# Supplementary material for: Rapid multiplex high resolution melting method to analyze inflammatory related SNPs in preterm birth
Source: BMC Res Notes. 2012 Jan 26;5:69. doi: 10.1186/1756-0500-5-69 (PMC3298535; doi:10.1186/1756-0500-5-69)
Supplement: Additional file 2 — Subset of DNA samples genotyped by different techniques. Genotype assignment for each analyzed SNP by Multiplex HRM, RFLP and sequencing. [file 1756-0500-5-69-S2.PDF]

Additional file 2: Subset of DNA samples genotyped by different techniques

TLR4 rs4986790	Genotyping technique		
	**HRM**	**RFLP**	**SEQ**
BC001	AA	AA	
BC002	AA	AA	AA
BC004	AA	AA	
BC006	AA	AA	
BC010	AA	AA	
BC011	AA	AA	AA
BC013	AA	AA	AA
BC029	AA	AA	AA
BC042	AA	AA	AA
BC054	AA	AA	AA
BP007	AG	AG	AG
BP008	AA	AA	
BP009	AA	AA	
BP014	AG	AG	AG
BP045	AG	AA	AA
BP060	AG	AG	AG
BP001	AA	AG	AA
BP028	AA	AG	AA
BP082	AA	AG	AA

IL6 rs1800795	Genotyping technique		
	**HRM**	**RFLP**	**SEQ**
BC100	GG	GG	GG
BC012	GC	GC	
BC013	GG	GG	
BC015	GC	GC	GC
BC016	GC	GC	
BC017	GG	GG	
BC018	GG	GG	GG
BC022	GC	GC	
BC025	GG	GG	
BC026	GC	GC	
BC027	GG	GG	
BC028	GG	GC	GC
BC029	GC	GC	GC
BC032	GG	GC	GC
BC035	GC	GC	GC
BC216	GG	GG	GG

IL1B rs16944	Genotyping technique		
	**HRM**	**RFLP**	**SEQ**
BC001	AG	AA	AG
BC002	AG	AG	AG
BC003	GG	GG	
BC004	AG	AG	AG
BC005	AG	AG	AG
BC006	GG	GG	GG
BC007	AA	AA	
BC008	GG	GG	GG
BC010	GG	GG	GG
BC011	GG	GG	GG
BC012	AG	AG	AG
BC015	AA	AA	AA
BC100	AG	AG	AG
BP059	GG	GG	GG
BC114	AG	AG	AG
BP001	GG	GG	GG

IL12RB rs375947	Genotyping technique		
	**HRM**	**RFLP**	**SEQ**
BC100	AA	AA	AA
BC002	AA	AA	
BC011	AA	AA	
BC029	AA	AA	
BC032	AA	AA	
BC042	AA	AA	
BC054	AA	AA	
BP007	AA	AA	
BP008	AA	AA	AA
BP009	AA	AA	AA
BP010	AA	AA	AA
BP011	GG	AA	AA
BP013	AA	AA	AA
BP014	AA	AA	
BC217	AA	AA	AA
BC001	AG	AG	AG
BC004	AA	AG	
BC006	AG	AG	AG
BC018	AG	AG	AG
BC039	AG	AG	
BC005	GG	GG	GG
BC015	GG	GG	
BC022	GG	GG	GG
