# Supplementary figures and images for: Rapid multiplex high resolution melting method to analyze inflammatory related SNPs in preterm birth
Source: BMC Res Notes. 2012 Jan 26;5:69. doi: 10.1186/1756-0500-5-69 (PMC3298535; doi:10.1186/1756-0500-5-69)

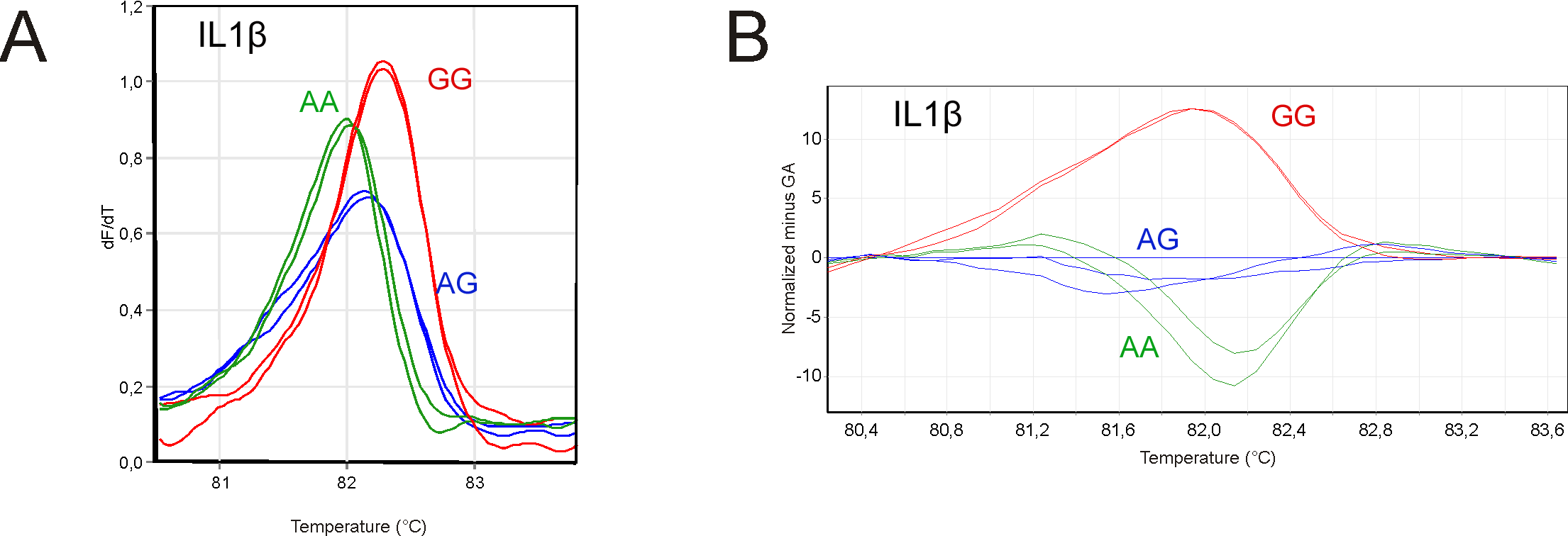

Supplement: Additional file 3 — Figure S1. A) Melting profile for rs16944 (IL1β). Red: Homozygote GG. Blue: Heterozygote AG. Green: Homozygote AA. B) Normalized difference plot for rs16944 (IL1β), genotype AG as a reference. [file 1756-0500-5-69-S3.TIFF]
